# Tailoring the Blast Exposure Conditions in the Shock Tube for Generating Pure, Primary Shock Waves: The End Plate Facilitates Elimination of Secondary Loading of the Specimen

**DOI:** 10.1371/journal.pone.0161597

**Published:** 2016-09-07

**Authors:** Matthew Kuriakose, Maciej Skotak, Anthony Misistia, Sudeepto Kahali, Aravind Sundaramurthy, Namas Chandra

**Affiliations:** Center for Injury Biomechanics, Materials and Medicine (CIBM^3^), Department of Biomedical Engineering, New Jersey Institute of Technology, Newark, NJ, 07102–1982, United States of America; University of Florida, UNITED STATES

## Abstract

The end plate mounted at the mouth of the shock tube is a versatile and effective implement to control and mitigate the end effects. We have performed a series of measurements of incident shock wave velocities and overpressures followed by quantification of impulse values (integral of pressure in time domain) for four different end plate configurations (0.625, 2, 4 inches, and an open end). Shock wave characteristics were monitored by high response rate pressure sensors allocated in six positions along the length of 6 meters long 229 mm square cross section shock tube. Tests were performed at three shock wave intensities, which was controlled by varying the Mylar membrane thickness (0.02, 0.04 and 0.06 inch). The end reflector plate installed at the exit of the shock tube allows precise control over the intensity of reflected waves penetrating into the shock tube. At the optimized distance of the tube to end plate gap the secondary waves were entirely eliminated from the test section, which was confirmed by pressure sensor at T4 location. This is pronounced finding for implementation of pure primary blast wave animal model. These data also suggest only deep in the shock tube experimental conditions allow exposure to a single shock wave free of artifacts. Our results provide detailed insight into spatiotemporal dynamics of shock waves with Friedlander waveform generated using helium as a driver gas and propagating in the air inside medium sized tube. Diffusion of driver gas (helium) inside the shock tube was responsible for velocity increase of reflected shock waves. Numerical simulations combined with experimental data suggest the shock wave attenuation mechanism is simply the expansion of the internal pressure. In the absence of any other postulated shock wave decay mechanisms, which were not implemented in the model the agreement between theory and experimental data is excellent.

## Introduction

Exposure to shock waves is identified as the leading cause of Traumatic Brain Injury (TBI) in military personnel [1, 2]. The injuries associated with explosive detonation are classified into four different categories based on their etiology: 1) primary, caused by pure shock waves, 2) secondary, resulting from penetration of fragmentation (shrapnel) and other projectiles into the brain parenchyma, 3) tertiary, originating from impact with other objects, and 4) quaternary, caused by exposure to heat and toxic gases [3–5]. It appears mixed type of injuries are expected near the epicenter of a blast, while shock wave is far reaching, compared to the other TBI risk factors associated with explosive blast. Traveling over long-distances, the shock wave could thus be the sole source of injuries far from the explosion, making primary blast TBI (bTBI) particularly onerous [6]. Researchers have been attempting to replicate field-relevant shock waves in laboratory settings through the use of shock tubes in order to investigate the generation and propagation of shock waves and possible mechanisms of bTBI, also referred to as blast induced neurotrauma (BINT) [7, 8]. Increased focus on bTBI has resulted in intensified research efforts and a number of groups have opted to use compressed-gas driven shock tubes to study the etiology of blast injury [9–14]. All categories of blast injuries can occur in the field [15–17], and while it is challenging to isolate cases of pure primary blast injuries among military personnel, in order to study biological effects of shock waves in the laboratory, it is crucial that these generated experimental wave forms are free of any artifacts. To date, there is only a limited understanding of conditions affecting shock wave propagation inside of the shock tube, what crucial differences exist between testing inside versus outside of the shock tube, and how the end-effects affect the shock wave profile and propagation [18, 19].

**Fig 1:**
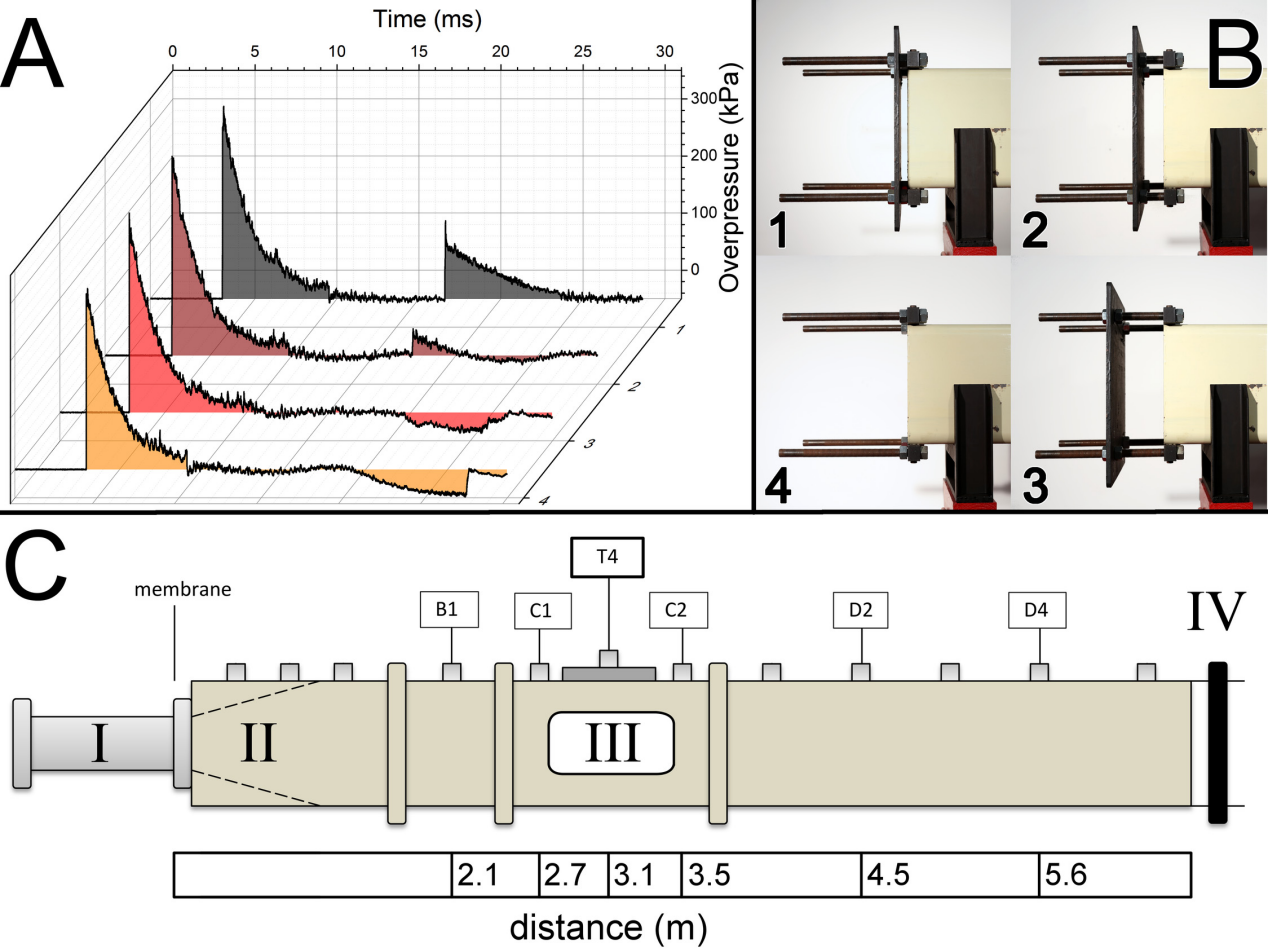
The representative incident shock wave profiles generated using helium as a driver gas and Mylar membrane (thickness of 1.016 mm), with accompanying secondary reflected shock and underpressure waves are presented (A). The profile of the secondary wave depends on the gap between the end plate reflector and the exit of the shock tube (B): 1. 0.625-inch, 2. 2-inch, 3. 4-inch, and 4. open end. C. Schematics of the 9-inch square cross section shock tube indicating the breech (I), transition (II), test section (III) and end plate (IV). Distribution of pressure sensor locations is also illustrated. Typically sensors B1, C1, T4, C2, D2 and D4 were used in our experiments to track the shock wave profile evolution along the entire length of the shock tube. The scale bar indicates the distance of specific sensor from the breech, i.e. Mylar membranes installation port.

Since their inception in 1899 [20], shock tubes have been widely used in a variety of research areas for studying phenomena which require extremely high temperatures and heating rates or occurring at extremely fast but controllable rates: high temperature chemical kinetics [21, 22], molecular spectroscopy [23], and to simulate interaction of plasma with Earth’s magnetosphere [24], just to name a few prominent examples. However, in the biomedical field, shock tubes found application as research tools relatively recently, with just a few existing peer-reviewed reports published pre 2010 [25–28]. Typically shock tubes share major design features, which utilize three essential components: driver (breech), driven (which includes the test section) and the end-reflector. However, the subtle differences in the design and operation of the tube have significant impacts on the resulting pressure history measured inside of the tube: volume of the breech, breech-to-test section diameter ratio, circle-to-square transition and length of the driven section. It is generally accepted that the shock wave closely resembling the Friedlander waveform (Fig 1) should be used for any experiments aiming at replicating field blast conditions [29, 30]. This waveform consists of a sharp, almost instantaneous rise in pressure (shock front) followed by exponentially decaying pressure (blast wind) [31]. It is critical to accurately characterize the amount of loading sustained by subjects (it is of particular importance for studies which use animal models) and to tailor specific pressure profiles via optimization of shock tube configuration. To the best of our knowledge, systematic experimental characterization of conditions inside of the shock tube received only limited attention [32, 33], in spite of vast theoretical and empirical evidence regarding the fate of the shock wave traveling on the outside of the shock tube [34, 35].

In this work, the characteristics of the propagating shock wave (peak overpressure, duration, impulse and velocity) and secondary waves were followed by measuring the pressure-time profiles at six sensors distributed along the tube. We demonstrate conditions inside of the shock tube can be controlled and secondary waves eliminated by careful adjustment of the end plate reflector gap; thus the specimen is exposed only to a single shock wave with well-defined characteristics. This work has also wider implications for the testing using shock tubes: our data indicate the end of the shock tube is less desirable specimen location, since the incident shock wave exiting the shock tube is inseparable from secondary waves, and thus always exposed to mixed mode loading.

## Materials and Methods

### The shock tube

The shock tube housed at the Shock Wave Testing facility at the Center for Injury Biomechanics, Materials and Medicine (CIBM^3^) at the New Jersey Institute of Technology has a 9” square cross section and a modular design with the following characteristics: 1) adjustable volume breech, 2) variable length transition section, 3) the 6 meter long test section, equipped with bullet-proof glass windows for high speed video observation of the specimen during the shock wave exposure, and 4) the reflector end-plate (Fig 1). In this study, the compressed helium was filled into the fixed volume breech, separated from the main body of the shock tube with Mylar membranes of three thicknesses (*vide infra*). The pressure inside the breech was continuously monitored using a WIKA A-10 sensor (0–340 atm range) and the burst pressure was recorded for all the tests.

### Overpressure measurement

We measured incident blast overpressure waves and secondary waves using a series of pressure sensors distributed along the shock tube (Fig 1C). A custom made LabView program running on in-house built data acquisition system based on National Instruments PXI-6133 S Series multifunction DAQ modules and PXIe-1082 PCI Express chassis was used for this purpose. Pressure sensors used in our experiments were PCB Piezotronics (Depew, NY) model 134A24. All data were recorded at 1.0 MHz sampling frequency and the typical acquisition time was 200 milliseconds.

### Experimental design

This study was designed as two factor experimental design, 4 x 3. Two experimental variables investigated in this study are: 1) the distance between the reflector plate and the end of the shock tube (four levels) and 2) shock wave intensity (three levels). The distance between the reflector plate and the end of the shock tube was adjusted as shown in Fig 1B. Four different gap lengths were used: 1) 0.625”, 2) 2” and 3) 4”, and 4) open end. The incident blast overpressure was controlled by adjusting the thickness of Mylar membranes sandwiched between the breech and expansion section. The thickness was adjusted by stacking individual membranes with thickness of 0.01 inches (0.254 mm). In this study we used three membrane thicknesses (0.02, 0.04, and 0.06 inches, denoted as 2, 4 or 6 membranes, respectively). All tests were performed using single fixed breech volume of 6.5 x 10^−3^ m^3^, and were repeated six times for each combination of experimental covariates. Additional tests were also performed with a fully closed shock tube (in triplicates for all three membrane thicknesses) to establish boundary conditions for reflected wave intensities and velocities.

### Numerical simulations

The propagation of blast waves are modeled in the shock tube environment. The air inside the shock tube through which the blast wave propagates is modeled using Eulerian elements. The size of the Eulerian domain corresponds to the physical dimensions of the shock tube used in the experiments (cross-section: 229 x 229 mm). The heterogeneous meshing of the shock tube was adopted, with fine mesh near the end and coarse mesh elsewhere, in order to decrease total number of elements in the model without sacrificing accuracy.

#### Solution scheme

The finite element (FE) model is solved using the nonlinear transient dynamic procedure with the Euler-Lagrangian coupling method (Abaqus 6.10). In this procedure, the governing partial differential equations for the conservation of mass, momentum, and energy, along with the material constitutive equations and corresponding equations defining the initial and boundary conditions are solved simultaneously. The Eulerian framework allows the modeling of highly dynamic events (e.g., shock) which would otherwise induce heavy mesh distortion. An enhanced immersed boundary method was used to provide the coupling between the Eulerian and the Lagrangian domains.

### Comparison with field blast waves

Comparison of experimental data (overpressure profile recorded by sensor at T4 location) with idealized field blast profiles were performed using ConWep 2.1.0.8. [36]. The blast wave profiles were simulated as hemispherical ground explosion which were generated by TNT charge with weight in the range 10–2000 kg in the standoff distance range of 2–25 m. Only these profiles falling within range of both BOP and impulse of experimental data were used.

### Statistical analysis

Data from experiments preformed at different experimental conditions (end plate gap) were pooled together in 3 subsets according to blast intensity (membrane thickness). Data was checked for normality using Ryan-Joiner test (similar to Shapiro-Wilk) in Mintab 17.0. Then multiple comparison two-tailed t-test was performed with Bonferroni correction and p < 0.003 was considered statistically significant. Power analysis was performed with GPower 3.1.9.2 software. All data are presented as mean and standard deviation.

## Results

### Blast Overpressure and Burst Pressure

**Fig 2:**
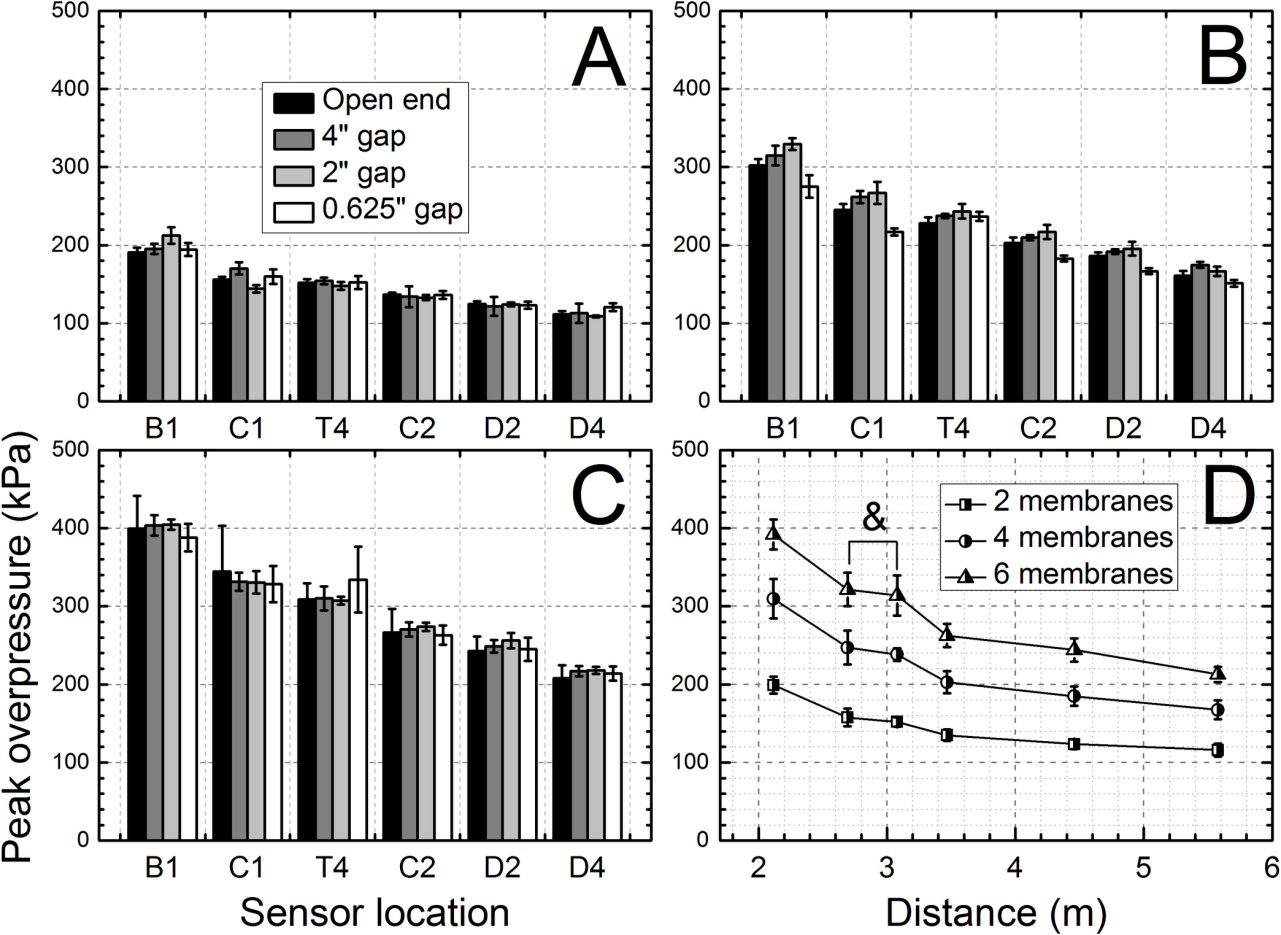
Peak overpressure inside of the shock tube as a function of sensor location and membrane thickness: A) 0.02”, B) 0.04”, and C) 0.06”. Peak overpressure values averaged among experiments preformed using the same Mylar membrane thickness (D): differences of average BOPs for sensors C1 and T4 are not statistically significant (marked with ampersand **&**, p > 0.05) in respective test groups.

Peak blast overpressures (BOP) for different membrane thicknesses and back plate conditions are presented in Fig 2. BOP increased with membrane thickness and shows a direct correlation with the burst pressure (the maximum pressure measured in the breech at the time of membrane rupture). The variations between the measured BOP in experiments where the same membrane thicknesses were used are relatively small for all four back plate conditions, as evidenced by narrow standard deviation values (Fig 2A–2C). The blast overpressure is found to be a linear function of the burst pressure (S1 Fig). The variations of the maximum burst pressure can be attributed to sample-to-sample differences in Mylar membrane mechanical properties, typical for this class of commercially available materials [37]. The variation in burst pressure increases with Mylar membrane thickness (i.e. number of membranes), as evidenced by increasing standard deviation (S1 Fig).

The general observed trend is that the peak BOP decreases as the shock wave travels down the tube, and the end plate configuration has no effect on the peak BOP, with an exception of peak overpressures recorded at sensor locations C1 and T4, for which differences are not statistically significant (p > 0.05, Fig 2D).

### Incident Shock Wave Velocities

**Fig 3:**
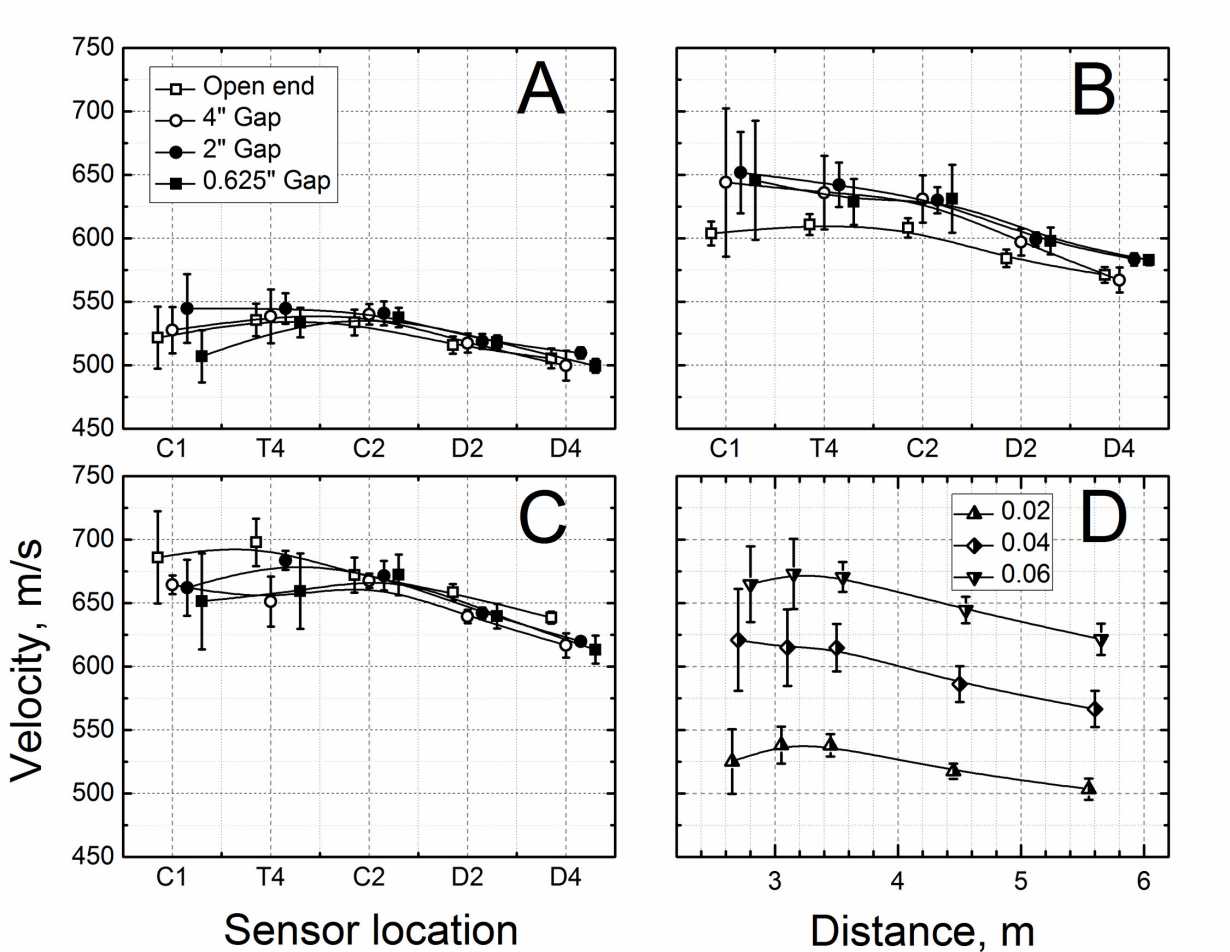
Calculated shock wave velocities at different sensor locations as a function of BOPs generated using Mylar membranes with thicknesses of: A) 0.02”, B) 0.04”, and C) 0.06”. Shock wave velocities were averaged for all experiments preformed using the same Mylar membrane thickness (D). Individual data points were horizontally shifted for clarity of presentation. The B1 sensor was used as a reference for all calculations.

The calculated values of the velocity of the shock front, traveling in the shock tube are presented in Fig 3. The shockwave velocities increase with the thickness of the Mylar membrane, but just as in the case of peak BOPs there appears to be virtually no difference between the shock velocities for varying back plate conditions. For all three Mylar membrane thicknesses used in these experiments the general observed trend is that the shock wave velocity decreases with the distance travelled in the shock tube: 540 to 500 m/s (deceleration: -16.4 m s^-2^, Fig 3A), 630 to 570 m/s (-22.9 m s^-2^, Fig 3B) and 680 to 620 m/s (-23.3 m s^-2^, Fig 3C), for the membrane thickness of 0.02, 0.04 and 0.06 inches, respectively. It is also clear that the shock wave velocity variation is higher the closer the measurement location is to the breech. The pooled data for all measurements show narrow standard deviations for the velocities for all sensor locations after the test section sensor T4 (C2, D2 and D4). The velocities remain unchanged until shock wave reaches sensor D2 (p < 0.005, power: >0.95 with respect to velocities at T4 and C2) for all three tested membrane thicknesses (Fig 3D).

### Impulse

**Fig 4:**
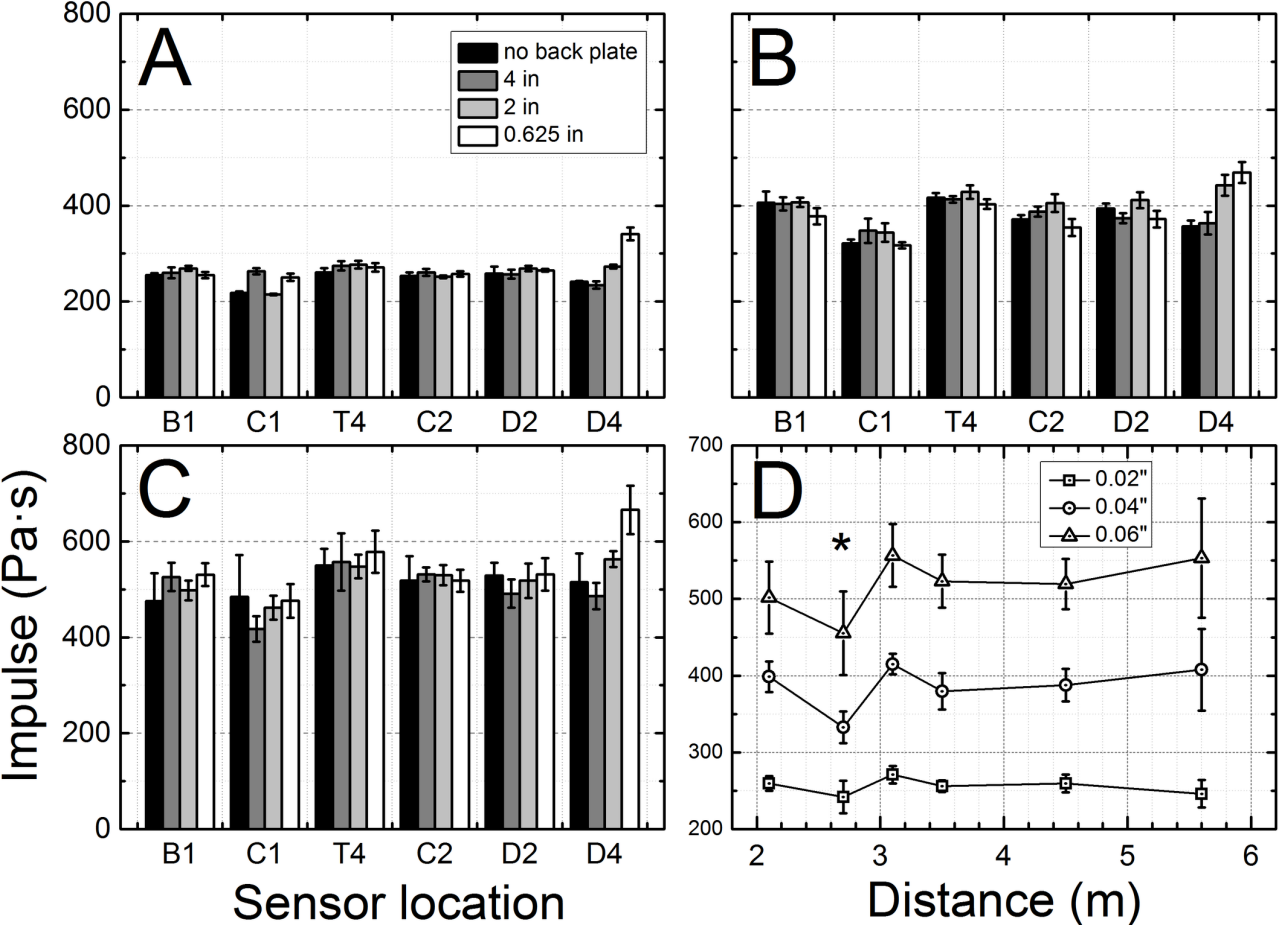
Positive phase impulse measured for the shock waves traveling inside of the shock tube as a function of sensor location and membrane thickness: A) 0.02”, B) 0.04”, and C) 0.06”. The average impulse of shock waves recorded for three respective Mylar membrane thicknesses used as a function of sensor location along the shock tube (D). Asterisk indicates impulse value recorded by the C1 sensor, which shows statistically significant difference in respective data sets (p < 0.003, power: >0.95).

The integrals of the shock wave overpressures versus time (impulse) generated using three different membrane thicknesses are depicted in Fig 4. In general, the impulse values increase with increased number of Mylar membranes (burst pressure) used (Fig 4A–4C). Within the same group the impulse variation along the length of the shock tube is relatively small, except for the C1 and D4 sensors. The most important differences are observed for the sensor D4, where the impulse is significantly higher for 0.625” end gap, when are compared to other end plate configurations (Fig 4A–4C).

The results of analysis of all pooled data revealed (Fig 4D) that differences between impulse values recorded at the C1 and T4 locations are statistically significant (p < 0.003, power: >0.95) when compared to other sensor locations. Similarly to BOP measurements the standard deviations tend to increase with increasing burst pressure (number of Mylar membranes used).Our experimental overpressure profiles recorded in the test section (T4 sensor location) match field explosion BOPs and impulses retrieved simulated using TNT charge weight of 19.8, 34.2 and 66.0 kg at standoff distance of 7.0–7.6 m for 2, 4 and 6 membranes, respectively (S1 Table and S2 Fig).

### Quantification of secondary positive and under-pressure waves

**Fig 5:**
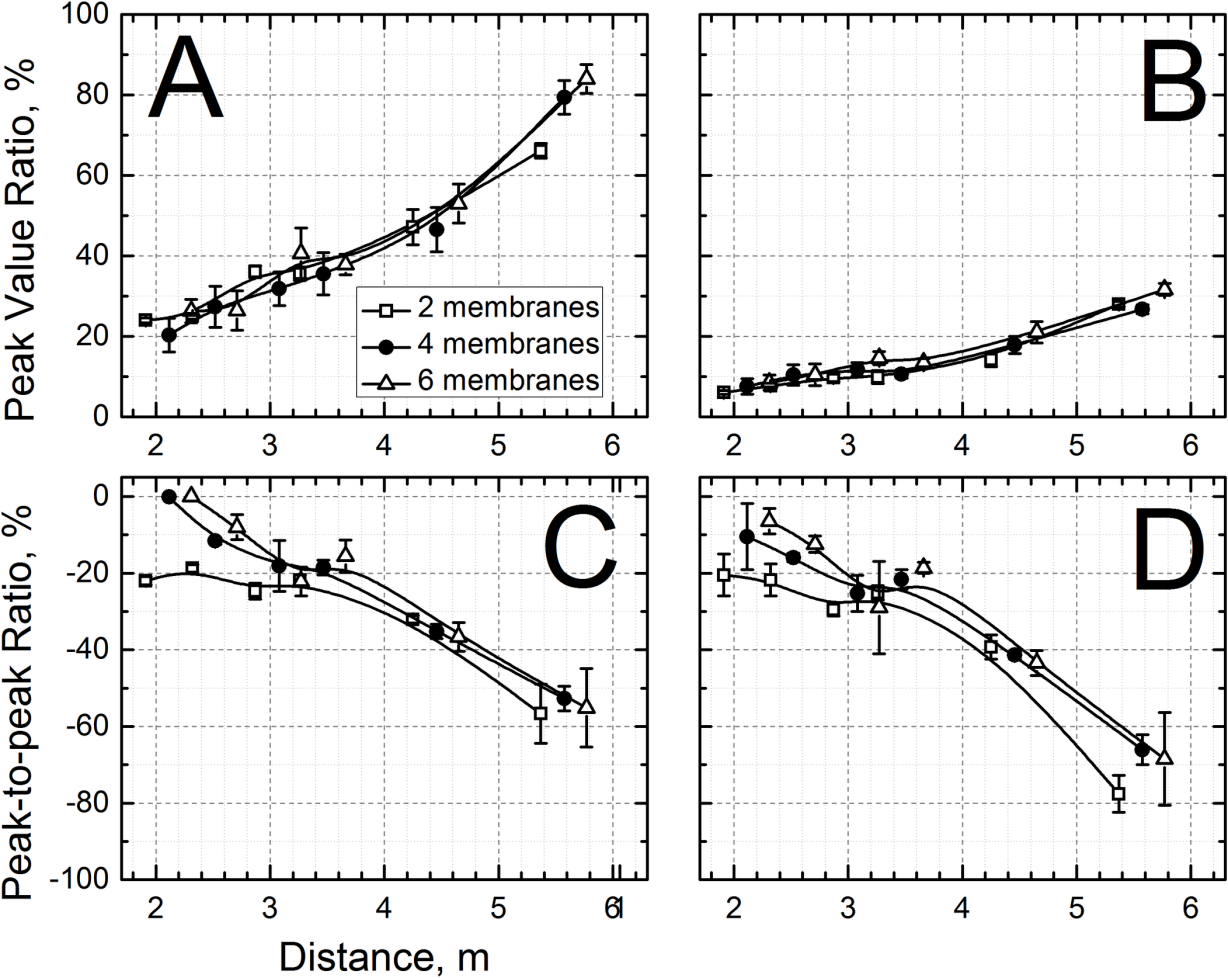
The ratios of peak overpressure between incident and reflected shock wave measured at different locations inside of the shock tube as a function of sensor distance from the breech and blast intensity: A) 0.625” gap, B) 2” gap between end of the shock tube and reflector plate. The ratios between incident peak overpressure and the lowest level (through) of measured reflected underpressure for blasts generated when the gap between the end of the shock tube and reflector plate was 4” (C) and with open end (D). The data points were horizontally shifted for clarity of presentation.

**Fig 6:**
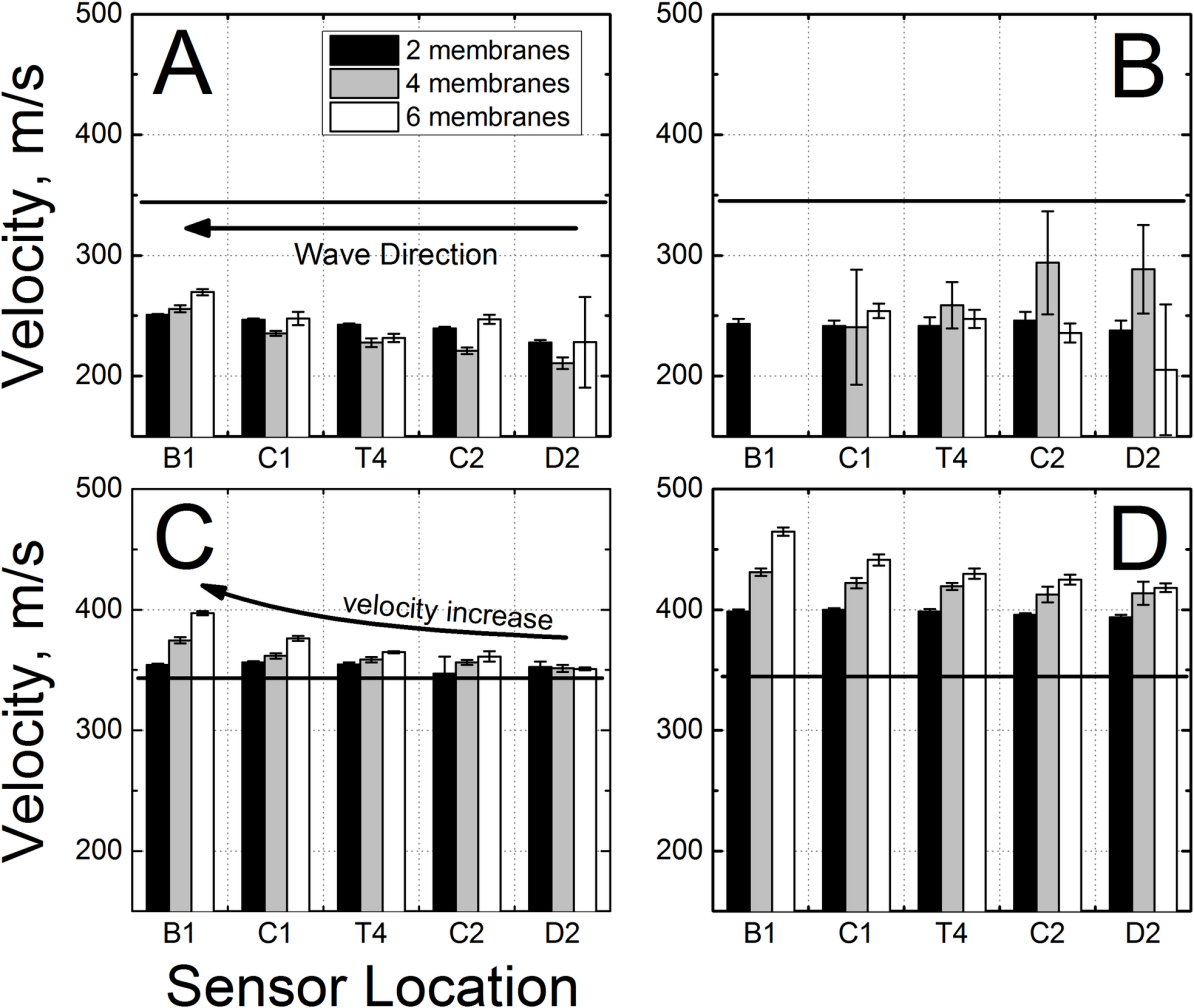
Velocities of reflected underpressure (A, B) and overpressure (C, D) waves generated with: A) open end, B) 4” gap, C) 2” gap and D) 0.625” gap. The straight arrow indicates the direction of the reflected waves’ propagation. The red and green horizontal lines indicate sound speed in the air. The velocity of reflected waves increases with the distance from the end of the shock tube, which is caused by increased helium (driver gas) concentration closer to the breech. The D4 sensor was used as reference.

The significance of the reflection waves and under-pressure waves on the loading of the test subject (in the test section at the T4 sensor location) is illustrated in Fig 5. When the end is fully open, the under-pressure wave that was generated had a minimum peak within 35% to 45% of the intensity of the original shock wave in the test section (as measured at T4 location). This means that the test subject (i.e. animal model) is being loaded with the original shock wave, and then shortly thereafter also by under-pressure wave, coming from the opposite direction. Implementing a back plate 4 inches away from the mouth of the shock tube decreases the under-pressure ratio to about 20% in the test section. Bringing the plate two inches closer completely eliminates the observed under-pressure waves, but causes a secondary reflected shock wave to travel back into the shock tube. In this configuration, the test subject is exposed to the original shock wave plus the additional reflection wave, with intensity of merely 10–15% of the original shock front, coming from the opposite direction. When the plate-to-shock tube gap is further decreased to 0.625 inches, the peak overpressure of incident to reflected shock wave ratio in the test section increases to 30–40% of the original shock front BOP. Velocities of reflected underpressure (open end and 4” gap) and overpressure waves (2” and 0.625” gap) are presented in Fig 6. The underpressure waves generated under all test configurations propagate inside of the shock tube with subsonic velocities (Fig 6A and 6B), while overpressure reflected waves retain supersonic but somewhat diminished velocities (Fig 6C and 6D).

### Optimization of end plate gap

**Fig 7:**
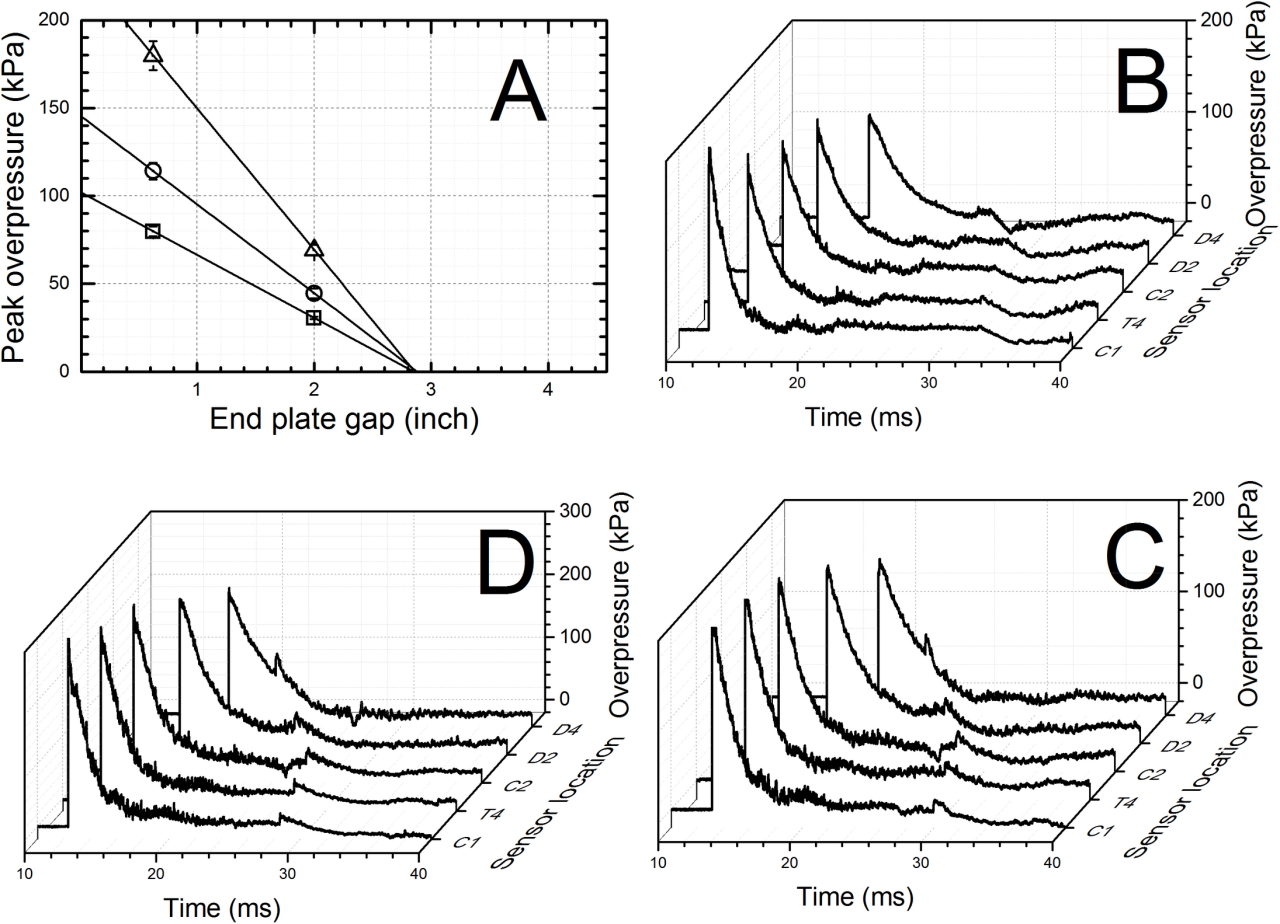
The optimization of the end plate to the shock tube gap distance. A. Reflected peak overpressure values measured at the D4 location for three different membrane thicknesses (0.02, 0.04 and 0.06 inch) and two different end plate gap sizes (0.625 and 2.0 inches) were used to identify the optimal gap size, i.e. the point on the plot where all linear functions converge (*x*_*0*_ = 2.85 inch). Overpressure profiles recorded using optimized end plate position at three different blast intensities generated using: B. 0.02”, C. 0.04” and D. 0.06”.

In this work we demonstrate control of the loading conditions inside of the shock tube can be easily achieved by the optimization of the end plate gap distance. The exposure of the specimen can be limited to a single shock wave without additional secondary over- or underpressure waves by optimization of the end plate gap distance. For this purpose we ran a series of tests generating shock waves with three different magnitudes. The shock tube end plate was set in the reflected shock wave regime (0.625” and 2” gap). Three linear functions were generated for both configurations (Fig 7A) and it turned out all these functions converge in a single point at 2.8 inches. The optimized end plate gap was then used to verify our predictions and as can be seen in Fig 7B–7D there are no secondary artifacts in the test section as measured by sensor located at T4.

### Numerical simulations

**Fig 8:**
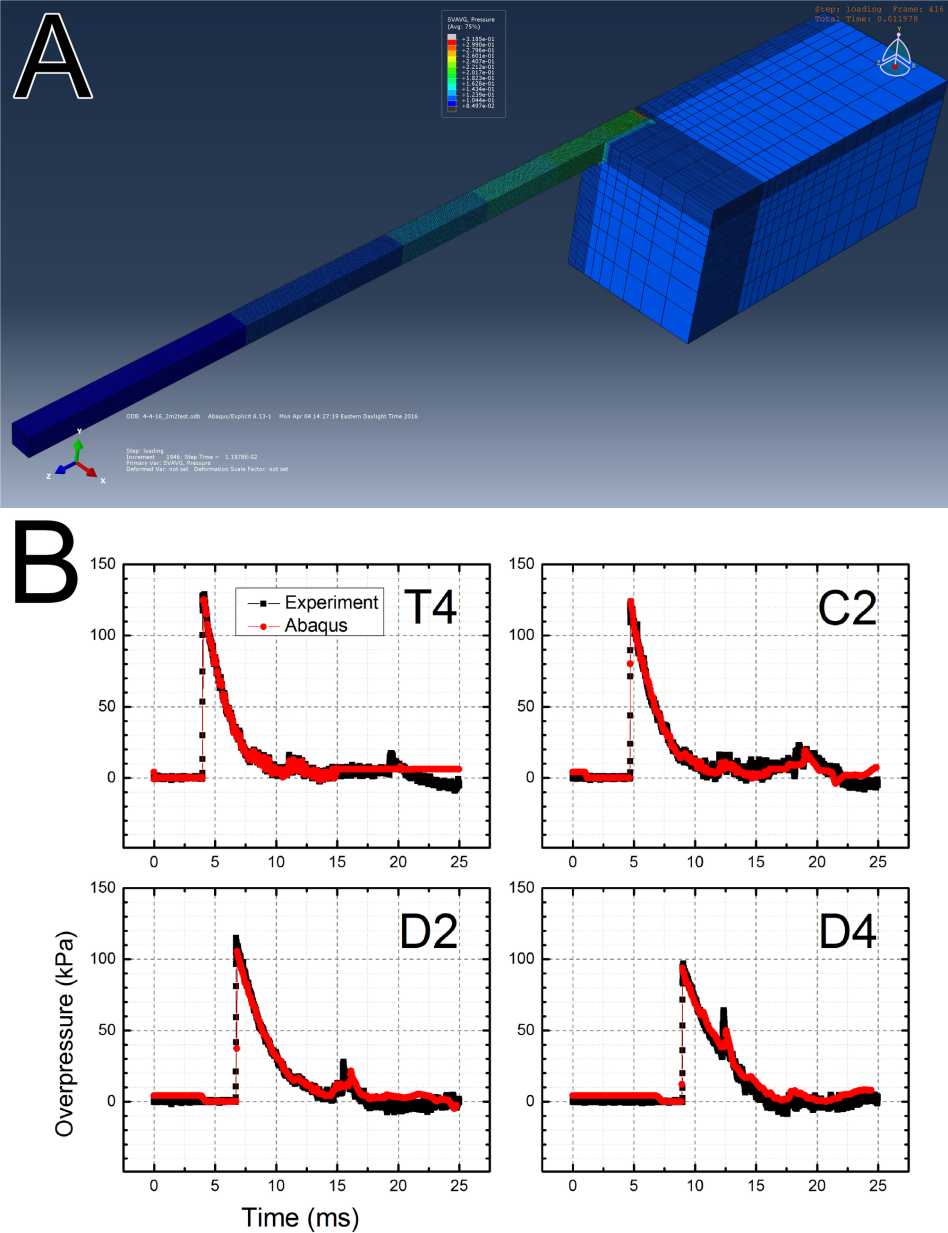
Numerical simulations: A) isometric view of the full scale model of the 9 inch square cross section shock tube, B) comparison of pressure traces recorded experimentally and obtained as results of numerical simulations with Abacus software for shock wave generated using 0.020” thick Mylar membrane and 2 inches end plate gap. Input feed for simulations was composed using initial 15 ms of the incident overpressure recorded by T4 sensor and 10 ms of baseline signal. This was done to eliminate secondary loading waveform from input data, which leads to erroneous calculations.

We have also performed numerical simulations of the shock wave propagating in the tube (Fig 8). The data recorded by the T4 sensor was used as the input for all three membrane thicknesses used in our experimental design. A representative example of results is presented in Fig 8B for pressure history of a shock wave generated using Mylar membrane with thickness of 0.020” and 2 inch end plate gap (other results are presented in S3–S7 Figs). Overall the results between simulations and experiments agree well. It would appear all characteristic features (peak overpressure, duration, and velocity) are replicated with good accuracy and fidelity for incident as well as for secondary waves.

### Experimental x-t diagram

**Fig 9:**
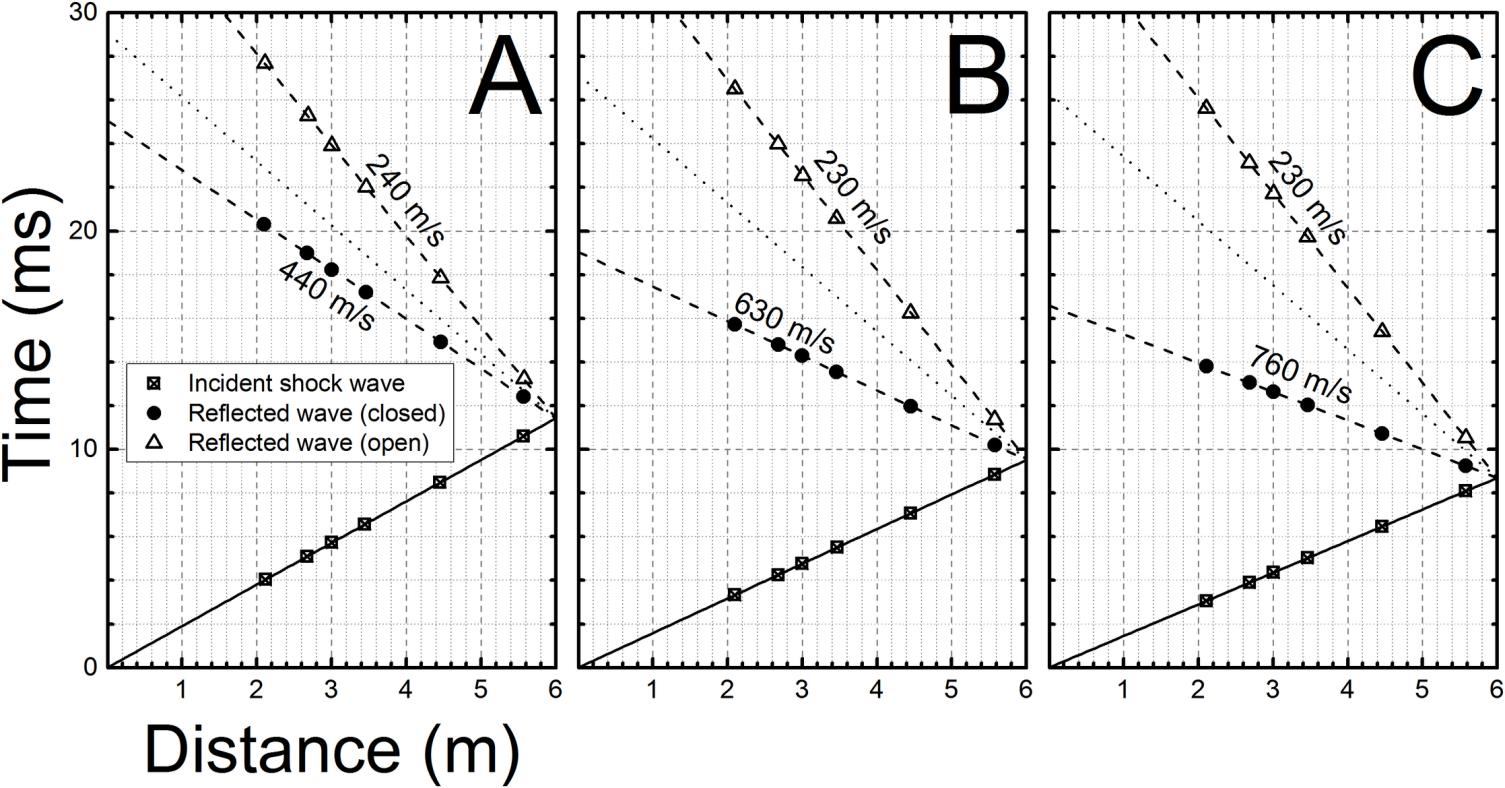
The distance-time (x-t) diagrams for incident and reflected waves based on experimental arrival times obtained at membrane thicknesses of: A) 0.02, B) 0.04, and C) 0.06 inches, respectively. The traces representing reflected waves are from experiments when the tube was fully closed and the underpressure waves observed when no endplate was present. Speed of sound for reflected wave domain is marked as dotted line.

In order to establish boundary conditions for the x-t diagram we performed measurements of shock wave traveling in the shock tube with closed end (S8 Fig). This configuration results in generation of the maximum reflected pressures in the shock tube, and we performed three replicates per each of three membrane thicknesses. The other extreme case is fully open end which results in the underpressure secondary wave, and comparison between these two cases (fully closed reflected waves and the fully open underpressure waves) is presented in Fig 9.

## Discussion

Compressed-gas driven shock tubes remain the most widely used and convenient method to generate controlled characteristics shock waves in laboratory setting [13, 38]. In spite of the popularity of this test device, the development of the blast wave along the length of the tube is not well understood, and clarification is required regarding the nature of loading conditions (presence of reflection and under-pressure waves) inside the shock tube. Our earlier work has clearly shown that in order to simulate primary blast conditions, the specimen have to be placed well inside the shock tube [19, 39]. However, compressive or expansive pressure waves can still affect the loading condition of the specimen. If these secondary waves are not properly extinguished they can be introduced after the original shock wave, and thus result in additional, potentially unwanted biomechanical load sustained by the test subject. The mitigation of secondary loading is thus of paramount importance for studying the effects of shock waves, especially for delineation of elementary mechanisms of bTBI. In this paper, we investigate the use of an end-reflector plate to modify the reflection and under-pressure waves that reenter the shock tube and resulting loading conditions.

The incident pressure measured in the shock tube decreases as the shock wave propagates towards the exit (Fig 2), independent of the test configuration used. However, there are no statistically significant differences in BOPs measured by C1 and T4 sensors (p > 0.05, marked with ampersand). This indicates the dynamics of shock wave generation: the expanding driver gas impact is decreasing until it reaches minimum at C1, since the volume expansion exceeds B1 sensor position (calculated using ideal gas conditions), thus the BOP registered by C1 sensor remains virtually constant between C1 and T4 sensors, which is then followed by BOP decay further downstream (C2, D2 and D4 sensors, Fig 2D). The calculated average velocities of shock waves generated using three different membrane thicknesses, are in line with these observations (Fig 3), i.e. the decrease in BOP is accompanied by respective decrease in shock wave velocity. The impulse measurements reveal more detailed picture of the dynamics of the system (Fig 4). While the impulse is diminishing in the driver gas affected region (B1 to C1) the impulse of ‘free’ shock wave is increasing (C1 to T1) and thus fully developed shock wave is present at T4 location (Fig 4D). Further downstream the impulse is not changing its value, but considering the BOP is steadily decreasing with accompanying decreased velocity it indicates the dissipation of energy of the shock wave. There are currently a number of competing theories which explain the decay of shock waves. The decay of spherical and cylindrical shock waves (3D and 2D waves encountered in the field conditions) is caused by purely geometrical factor as the intensity of the shock wave decreases with as an inverse of the square radius (distance) from the epicenter (1/r^2^, for shock waves separated from the source) or following linear relationship (1/r), respectively [40]. However, these two are not applicable to unidimensional waves generated in the shock tube. The viscous effects near the shock tube walls (wall friction, growth of the boundary layer) were proposed as explanation of observed attenuation of the shock wave intensity in the tube [41, 42]. However, these effects are considered important for shock tubes with relatively small diameters ranging from 0.3 to 3.8 cm [41], while in our case the diameter is much larger (9 in, or 22.86 cm) and thus these effects are less likely to be responsible for observed attenuation. The dusty medium was proposed as responsible for decay of the shock waves propagating in the shock tube [32, 33]. In our case this effect is negligible, since no appreciable sized particles were purposely introduced into the environment and typically the number of dust particles in the air is insufficient to be solely responsible for the intensity decay. The hypothesis suggesting the rarefaction wave following the incident shock wave was also proposed as a mechanism of shock wave dissipation [39, 43], but in our experimental data this type of waves were never observed. However, in our model used for numerical simulations the agreement between experimental data and theory is excellent (Fig 8). This model doesn’t include any of postulated mechanisms of shock wave attenuation discussed in this paragraph. Thus, it appears simple expansion of the pressure contained within the shock wave is solely responsible for observed attenuation.

The values of impulses at the D4 sensor location for both short gap (0.625” and 2”, Fig 4) end plate configurations are obscured by presence of secondary shock waves, which overlap with a primary wave (Fig 8B, S4 and S6 Figs). This illustrates the loading experienced by the specimen in that location, i.e. the loading is never a single primary shock wave, but a sum of primary and secondary loading. The nature of secondary loading will depend on the end configuration and it will be: 1) additional reflected shock wave with intensity dependent on end plate gap, or 2) underpressure wave (for sufficiently large gap and open end). Independently on the nature of the secondary loading the conditions experienced by the specimen mounted in the location are the most complex than these encountered in any other location in the shock tube. The specimen receives almost double the loading of the original shock wave (for tests conducted with 0.625 and 2 inch gap) or mixed mode loading of incident over- and secondary under pressure waves (for 4 inches gap and open end configuration).

The gap size between the reflector plate and the end of the shock tube governs the type of generated secondary wave, i.e. when it is below critical distance reflected overpressure waves are generated, however when the gap size is larger than the critical size, the under-pressure waves are created (Figs 1A, 5 and 6). The nature of the generated waves dictates their propagation speed: the under-pressure waves generated during experimentation don’t exceed the speed of sound (Mach number = 1.0, marked as horizontal lines in plots presented in Fig 6), which is in agreement with existing knowledge [44]. The reflected waves for the shortest end-plate gap travelled faster than the speed of sound, but were slower than the original shock front (Figs 3, 6 and 9). Moreover, we observed an increase in velocity with the time of propagation for all reflected positive pressure shock waves. This phenomenon is caused by increased concentration of the driver gas (helium) in the part of the shock tube closer to the breech (sound speed in helium is 1007 m/s, while it is 343 m/s in air at 20°C) shortly after the membrane burst. Also, it should be noted that these reflected over- and under-pressure waves travel in the opposite direction of the original shock wave.

The calculated velocities of the underpressure and reflected shock waves illustrate the impact of the driver gas has on the dynamics of the shock wave propagation. As the compressed helium ruptures the membrane, the original shock wave travels into the body of the shock tube, and the driver gas is diffusing towards the test section. The reflected wave traveling towards the breech is propagating in the mixture of air and helium, and hence reflected shock wave velocity gradually increases. This effect is more pronounced for shots with six membranes (0.06” thickness), because higher helium pressure is necessary to cause membrane rupture (average burst pressure was 250, 490 and 730 psi, for membrane thickness of 0.02, 0.04 and 0.06 inch, respectively, S1 Fig). The velocity of reflection waves is decreased compared to the original shock front, because of energy loss after the reflection (and the portion of the blast that escapes through openings at the mouth of the tube).

The reflection and under-pressure ratios shown in Fig 6 demonstrate the effect of positioning an end-reflector plate at the end of the shock tube and potential additional loading that a test subject may sustain due to plate positioning or its absence. In order to minimize the loading caused by secondary waves reentering the shock tube, the end-reflector position has to be optimized to find the position where the action of over- or underpressure waves traveling back inside of the shock tube is minimized or completely eliminated. This can be achieved by running a series of calibration experiments at different incident shock wave intensities and end plate gap distances. However, this knowledge must be coupled with proper positioning of the specimen far away from the end of the shock tube to eliminate any possibility of end effects on the tests specimen.

## Conclusions

In this work we have systematically evaluated means to eliminate unwanted secondary artifacts in order to obtain pure shock wave waveform in the test section inside of the shock tube (T4 location). It is of particular importance for the study of effects of shocks on biological systems, with particular emphasis on the brain. Application of reflector end plate positioned within optimized perimeter resulted in elimination of virtually all secondary positive and negative pressure waves. Our results indicate the end of the shock tube is less favorable location to test specimen due to complexity of loading conditions. Incident shock wave exiting shock tube results in formation of underpressure waves, while reflected pressure waves are created when the end is fully closed or the reflector plate is at relatively short distance from the muzzle. Numerical simulations were used to corroborate the findings and combined with analysis of experimental data these results indicate shock wave decompression as plausible decay mechanism. The tests done in this study should serve as a strong starting point for researchers attempting to ensure accurate loading of test subjects in a compressed-gas driven shock tubes.

## Supporting Information

S1 Fig Burst pressure as a function of Mylar membrane thickness (left panel) and linear fit of the experimental data (right panel).
(PNG)

S2 Fig Comparison of blast overpressure profiles obtained experimentally in the 9-inch square cross section shock tube (T4 sensor) and simulated using ConWep and corresponding to explosion of: a) 19.8 kg TNT at standoff distance of 7.0 m, b) 34.2 kg of TNT at standoff distance of 7.0 m, and c) 66.0 kg of TNT at standoff distance of 7.6 m.
(PNG)

S3 Fig The close up of the shock tube (end) and surrounding space used in the model for numerical simulations (top panel). Comparison of pressure traces recorded experimentally and obtained as results of numerical simulations with Abacus software for shock wave generated using 0.020” thick Mylar membrane and 4 inches end plate gap. Input feed for simulations was composed using initial 15 ms of the incident overpressure recorded by T4 sensor and 10 ms of baseline signal. This was done to eliminate secondary loading waveform from input data, which leads to erroneous calculations. Underpressure wave penetrating inside of the shock tube is clearly visible at 12 ms (D4), 15 ms (C2), 18 ms (D2), and 20 ms (T4).
(PNG)

S4 Fig Comparison of pressure traces recorded experimentally and obtained as results of numerical simulations with Abacus software for shock wave generated using 0.040” thick Mylar membrane and 2 inches end plate gap. Input feed for simulations was composed using initial 15 ms of the incident overpressure recorded by T4 sensor and 10 ms of baseline signal. This was done to eliminate secondary loading waveform from input data.
(PNG)

S5 Fig Comparison of pressure traces recorded experimentally and obtained as results of numerical simulations with Abacus software for shock wave generated using 0.040” thick Mylar membrane and 4 inches end plate gap. Input feed for simulations was composed using initial 15 ms of the incident overpressure recorded by T4 sensor and 10 ms of baseline signal. This was done to eliminate secondary loading waveform from input data.
(PNG)

S6 Fig Comparison of pressure traces recorded experimentally and obtained as results of numerical simulations with Abacus software for shock wave generated using 0.060” thick Mylar membrane and 2 inches end plate gap. Input feed for simulations was composed using initial 15 ms of the incident overpressure recorded by T4 sensor and 10 ms of baseline signal. This was done to eliminate secondary loading waveform from input data.
(PNG)

S7 Fig Comparison of pressure traces recorded experimentally and obtained as results of numerical simulations with Abacus software for shock wave generated using 0.040” thick Mylar membrane and 4 inches end plate gap. Input feed for simulations was composed using initial 15 ms of the incident overpressure recorded by T4 sensor and 10 ms of baseline signal. This was done to eliminate secondary loading waveform from input data.
(PNG)

S8 Fig Example pressure traces recorded along the shock tube (incident pressure) for closed end configuration shots at: A. 2, B. 4 and C. 6 membranes. Only incident shock wave and first reflected wave peak are presented for clarity.
(PNG)

S1 File Tabulated numerical values of raw experimental data used in Figs 2 to 9. (XLSX)

S1 Table The comparison of the characteristic parameters of the shock waves generated in the 9-inch square cross section shock tube by sensor at T4 location and blast wave profiles generated using ConWep. Average values and standard deviations are reported.
(PDF)
