# Supplementary figures and images for: Tailoring the Blast Exposure Conditions in the Shock Tube for Generating Pure, Primary Shock Waves: The End Plate Facilitates Elimination of Secondary Loading of the Specimen
Source: PLoS One. 2016 Sep 7;11(9):e0161597. doi: 10.1371/journal.pone.0161597 (PMC5014318; doi:10.1371/journal.pone.0161597)

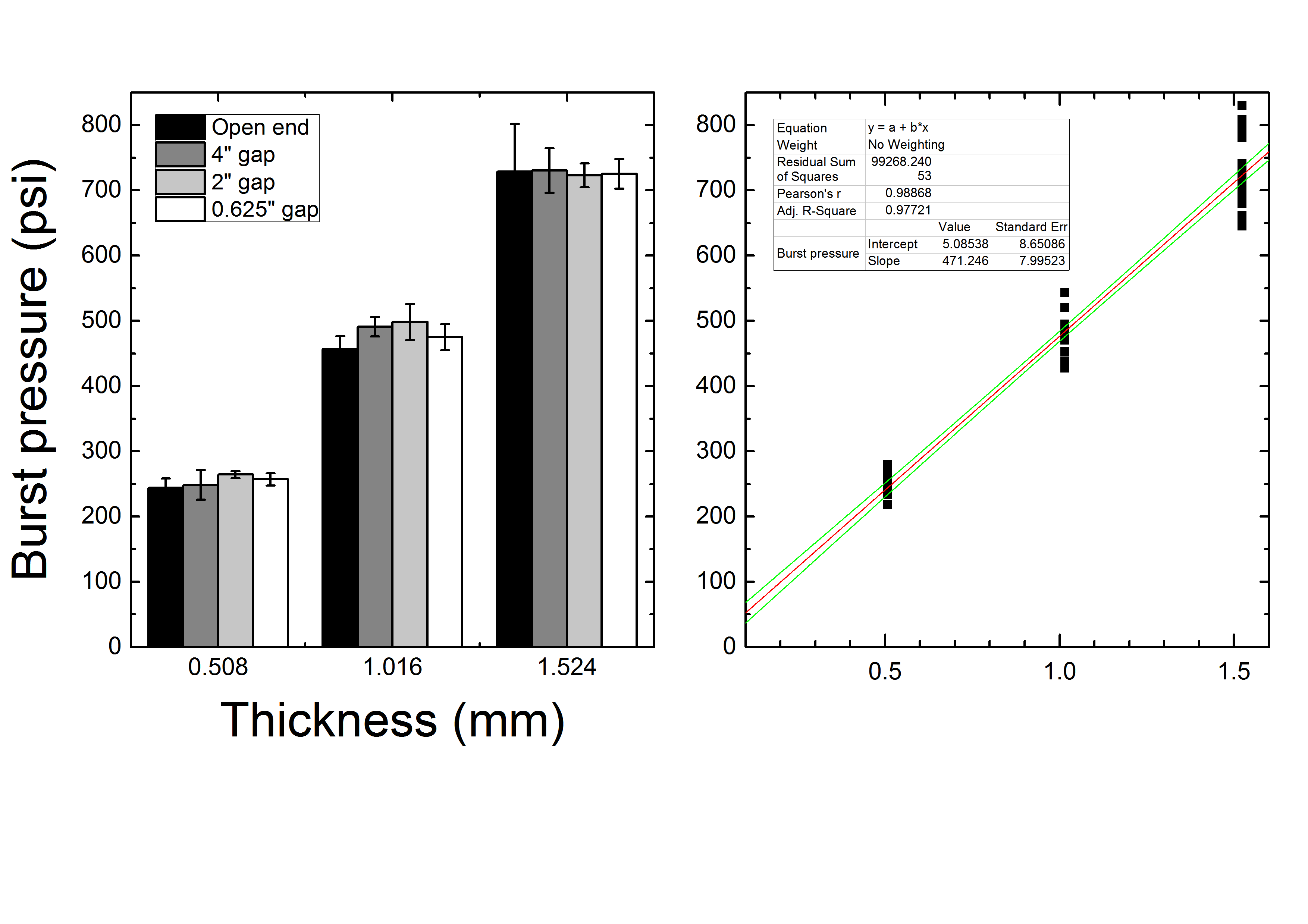

Supplement: S1 Fig — Burst pressure as a function of Mylar membrane thickness (left panel) and linear fit of the experimental data (right panel). (PNG) [file pone.0161597.s001.png]

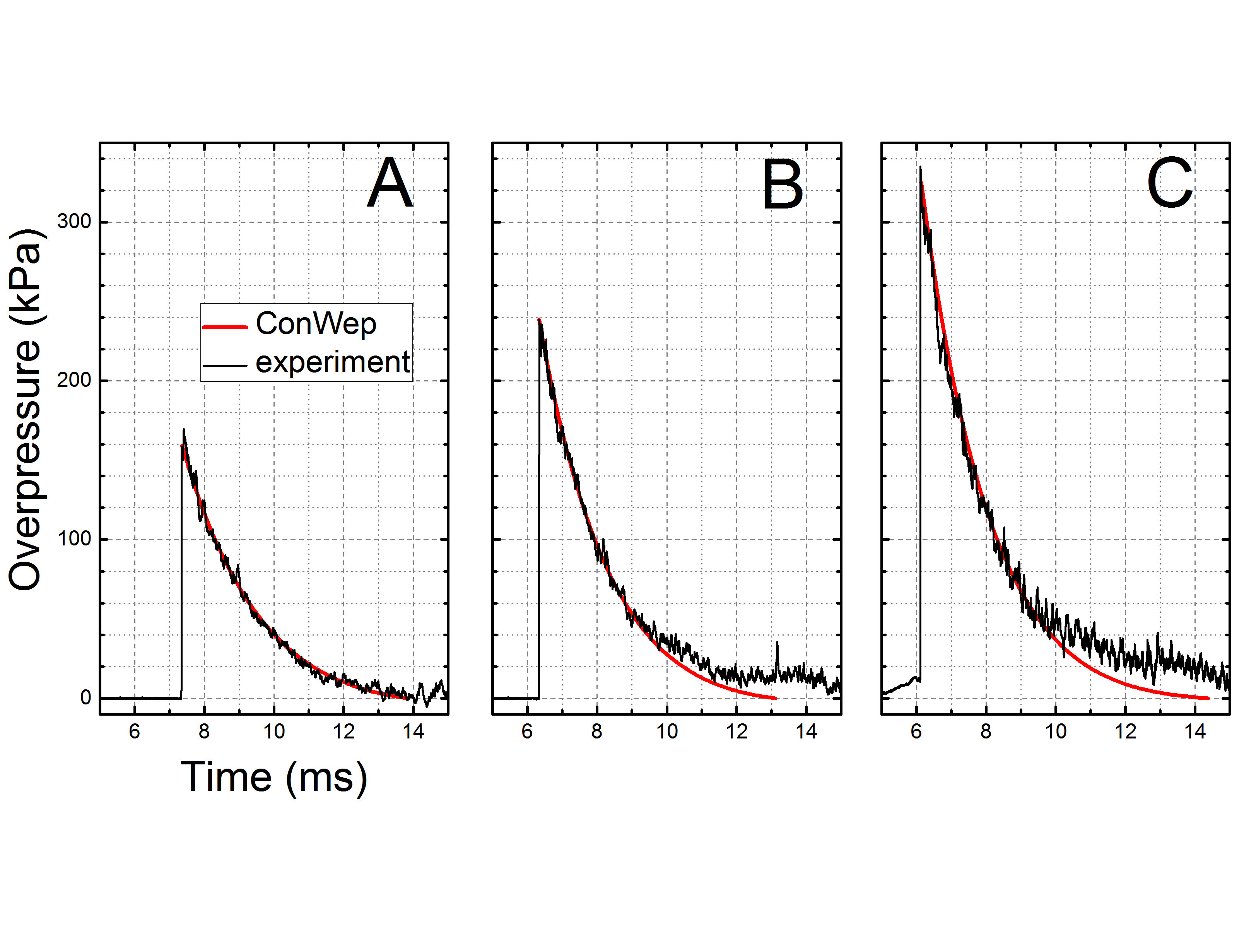

Supplement: S2 Fig — Comparison of blast overpressure profiles obtained experimentally in the 9-inch square cross section shock tube (T4 sensor) and simulated using ConWep and corresponding to explosion of: a) 19.8 kg TNT at standoff distance of 7.0 m, b) 34.2 kg of TNT at standoff distance of 7.0 m, and c) 66.0 kg of TNT at standoff distance of 7.6 m. (PNG) [file pone.0161597.s002.png]

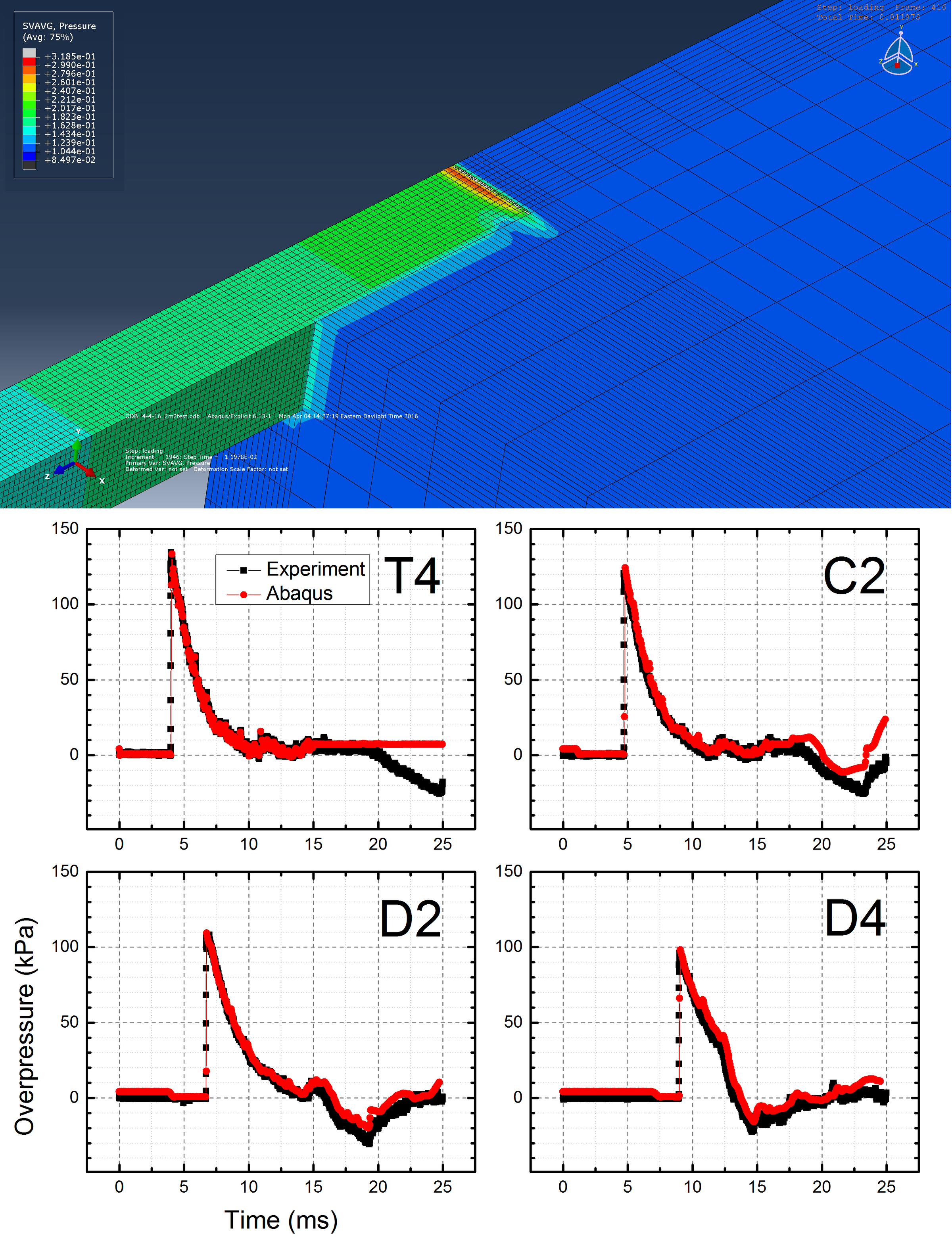

Supplement: S3 Fig — Comparison of pressure traces recorded experimentally and obtained as results of numerical simulations with Abacus software for shock wave generated using 0.020” thick Mylar membrane and 4 inches end plate gap. Input feed for simulations was composed using initial 15 ms of the incident overpressure recorded by T4 sensor and 10 ms of baseline signal. This was done to eliminate secondary loading waveform from input data, which leads to erroneous calculations. Underpressure wave penetrating inside of the shock tube is clearly visible at 12 ms (D4), 15 ms (C2), 18 ms (D2), and 20 ms (T4). (PNG) [file pone.0161597.s003.png]

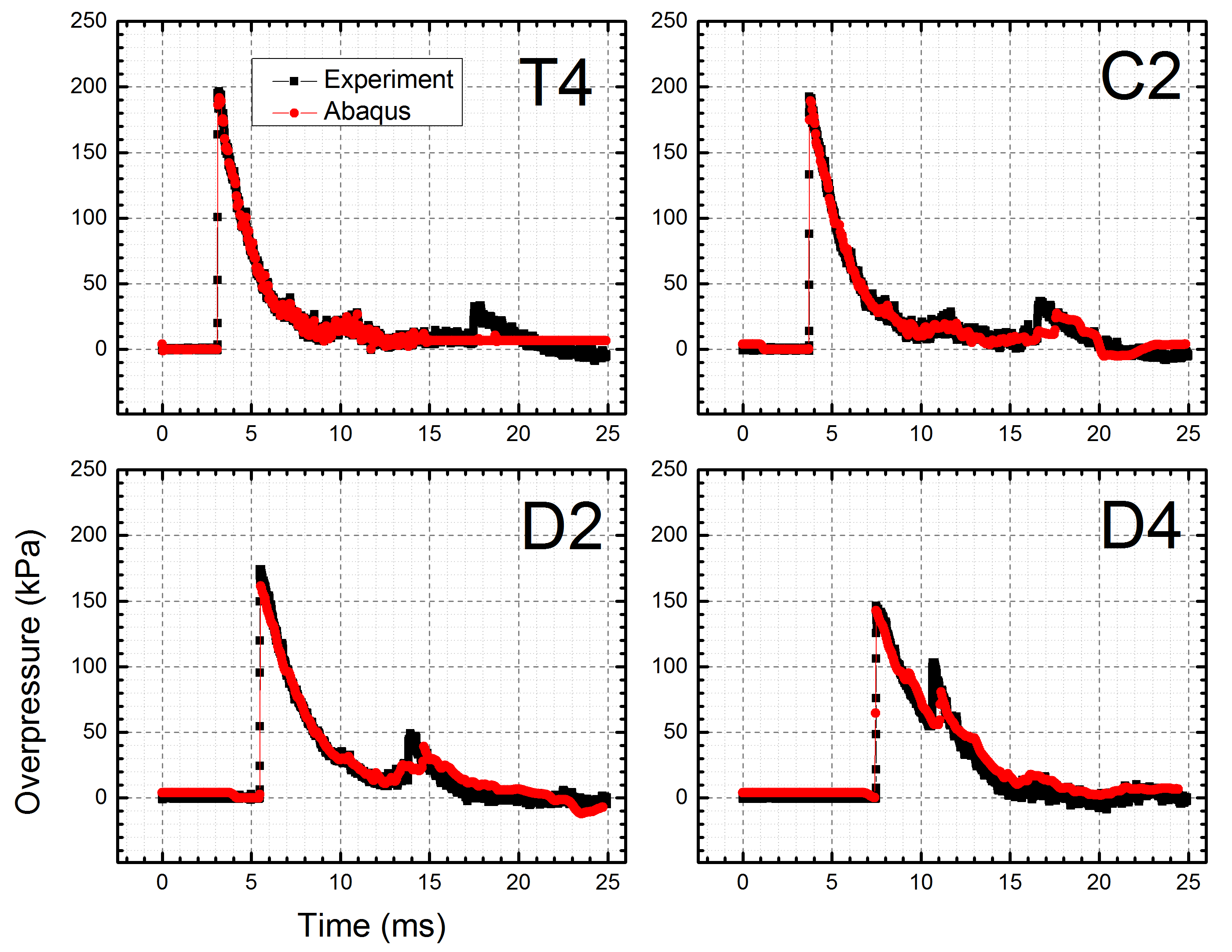

Supplement: S4 Fig — Input feed for simulations was composed using initial 15 ms of the incident overpressure recorded by T4 sensor and 10 ms of baseline signal. This was done to eliminate secondary loading waveform from input data. (PNG) [file pone.0161597.s004.png]

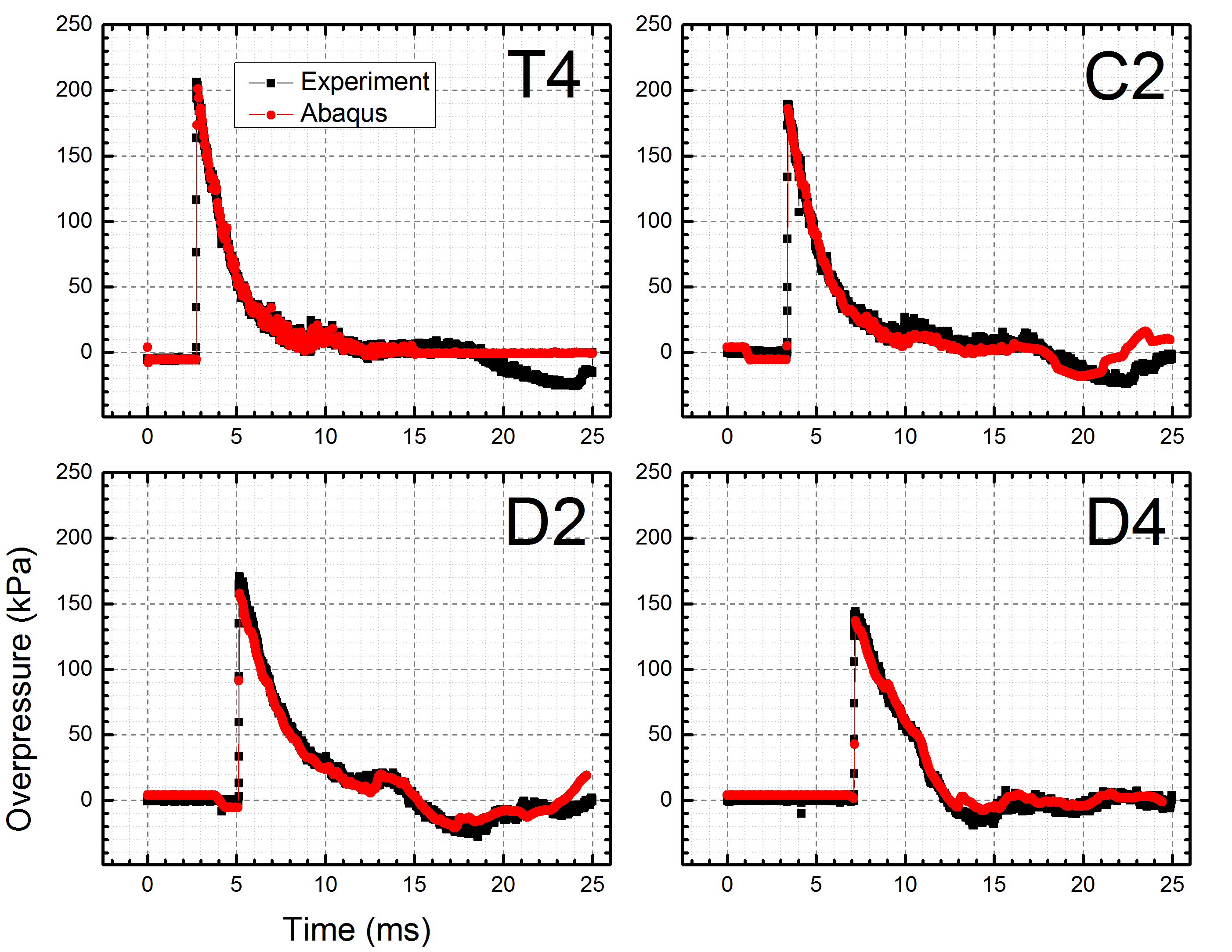

Supplement: S5 Fig — Input feed for simulations was composed using initial 15 ms of the incident overpressure recorded by T4 sensor and 10 ms of baseline signal. This was done to eliminate secondary loading waveform from input data. (PNG) [file pone.0161597.s005.png]

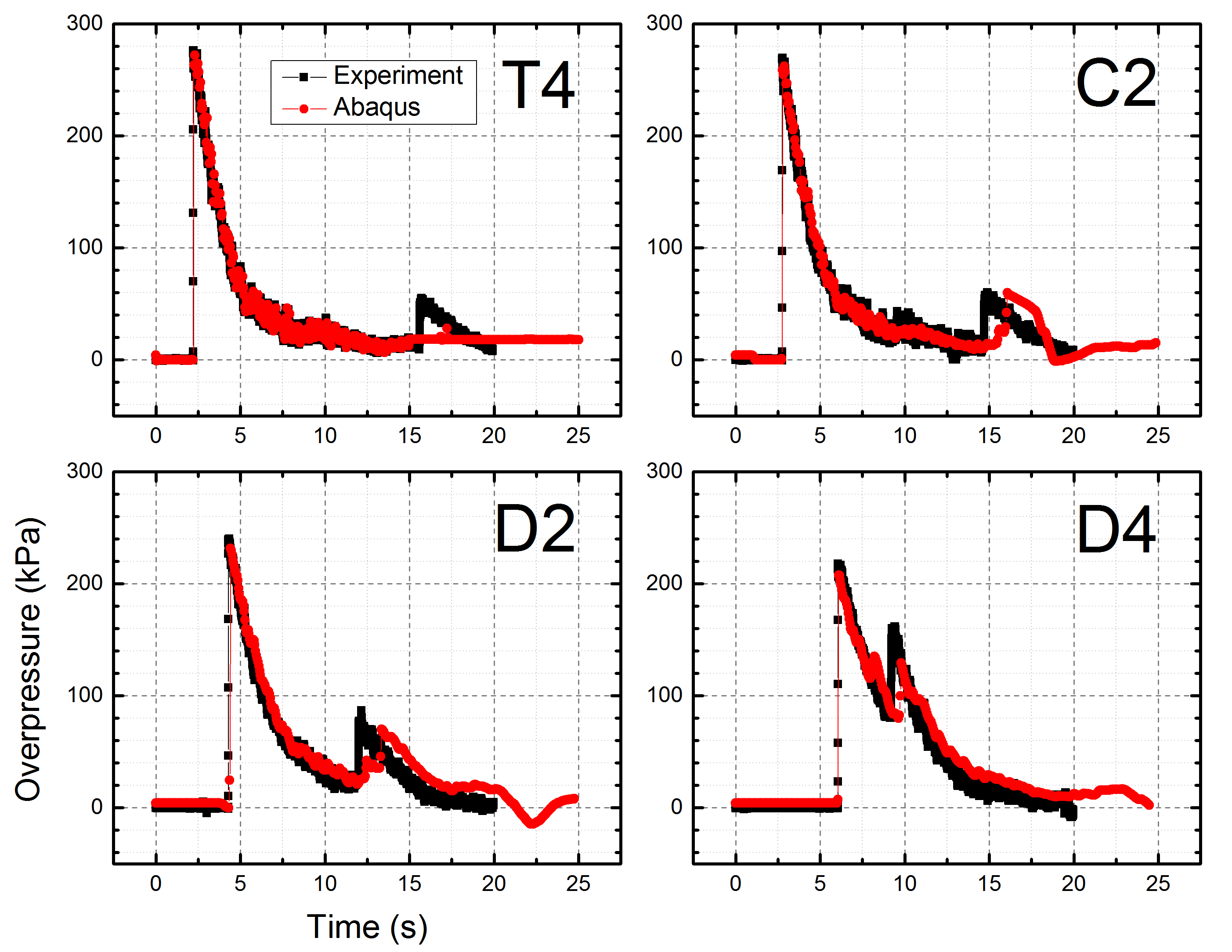

Supplement: S6 Fig — Input feed for simulations was composed using initial 15 ms of the incident overpressure recorded by T4 sensor and 10 ms of baseline signal. This was done to eliminate secondary loading waveform from input data. (PNG) [file pone.0161597.s006.png]

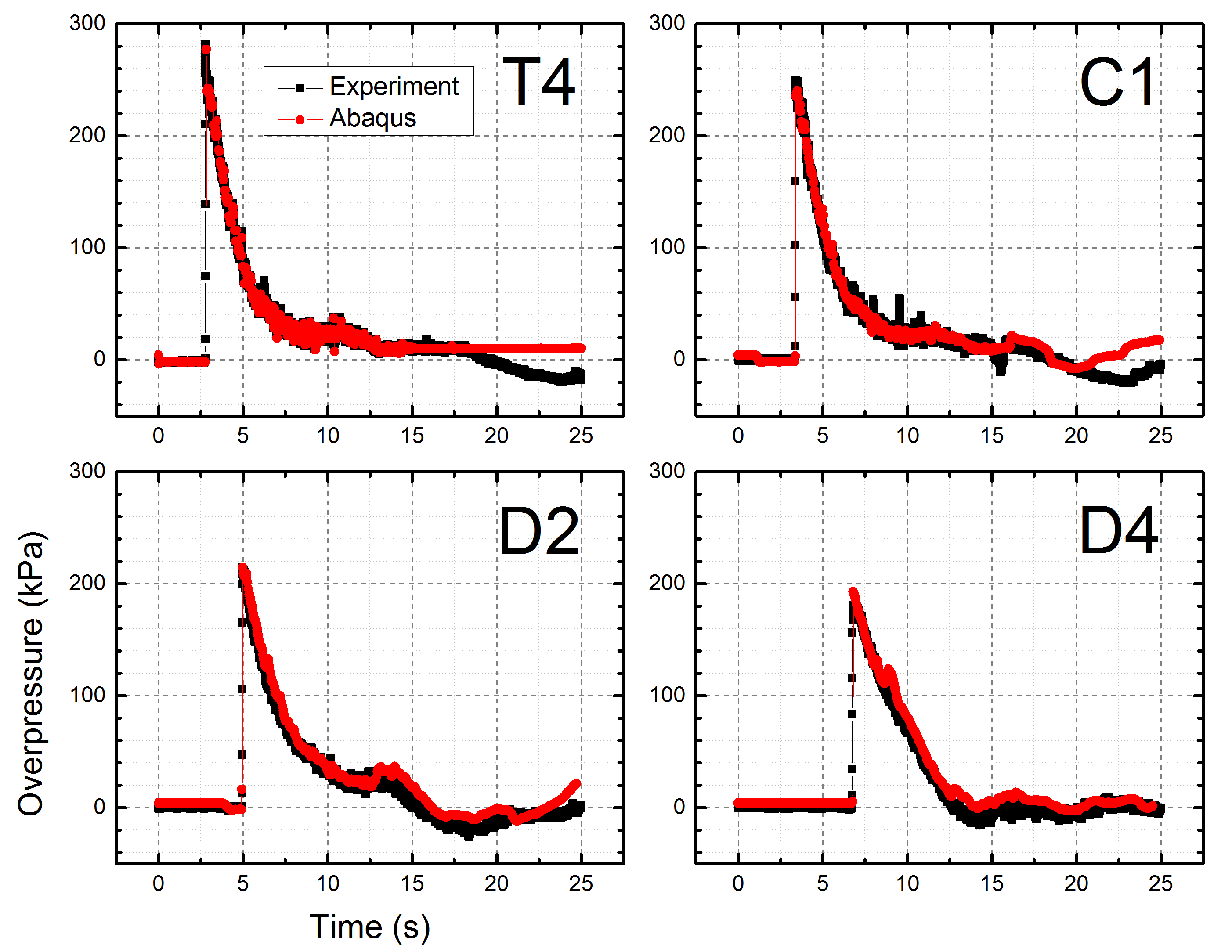

Supplement: S7 Fig — Input feed for simulations was composed using initial 15 ms of the incident overpressure recorded by T4 sensor and 10 ms of baseline signal. This was done to eliminate secondary loading waveform from input data. (PNG) [file pone.0161597.s007.png]

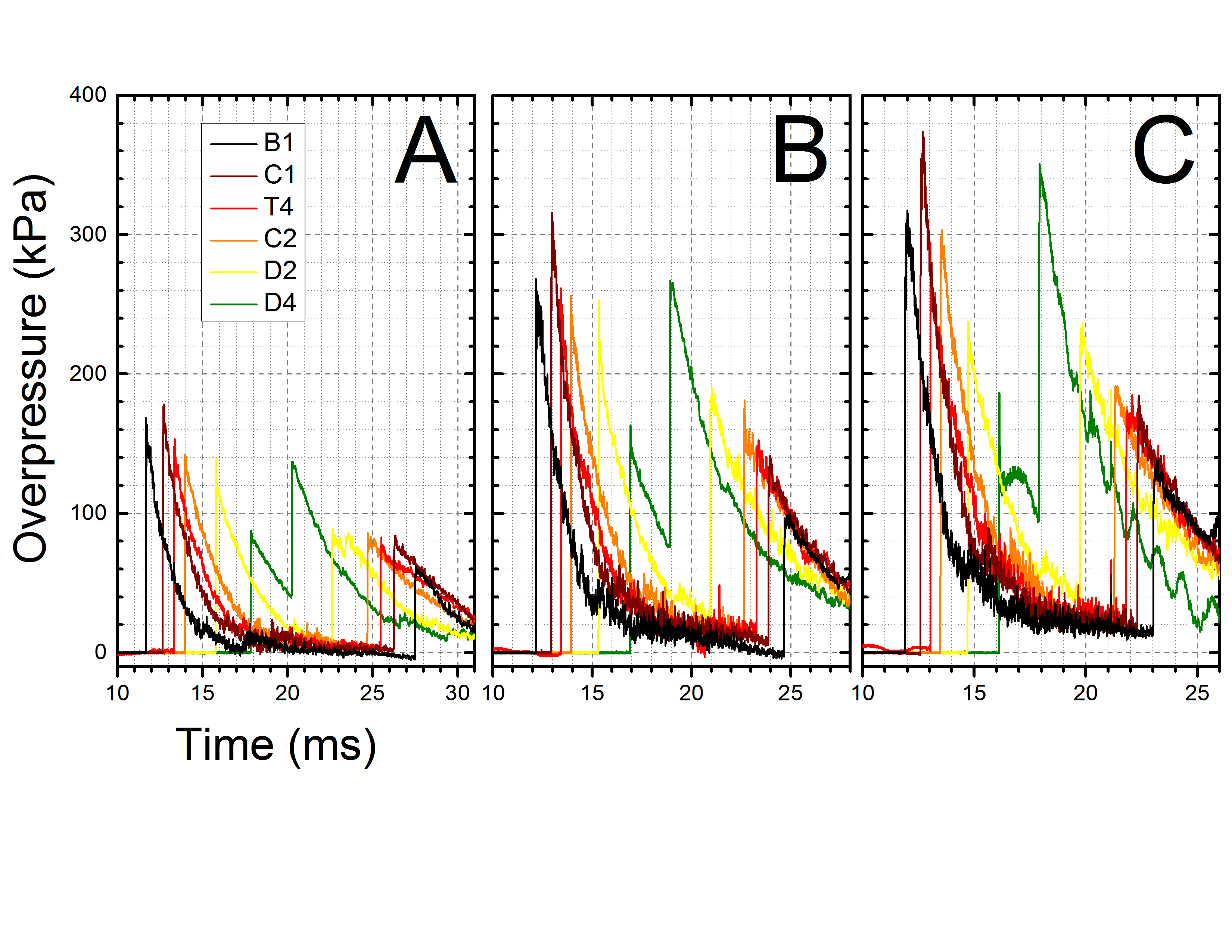

Supplement: S8 Fig — Example pressure traces recorded along the shock tube (incident pressure) for closed end configuration shots at: A. 2, B. 4 and C. 6 membranes. Only incident shock wave and first reflected wave peak are presented for clarity. (PNG) [file pone.0161597.s008.png]
