# Supplementary material for: Tailoring the Blast Exposure Conditions in the Shock Tube for Generating Pure, Primary Shock Waves: The End Plate Facilitates Elimination of Secondary Loading of the Specimen
Source: PLoS One. 2016 Sep 7;11(9):e0161597. doi: 10.1371/journal.pone.0161597 (PMC5014318; doi:10.1371/journal.pone.0161597)
Supplement: S1 Table — Average values and standard deviations are reported. (PDF) [file pone.0161597.s010.pdf]

	2 membranes		4 membranes		6 membranes	
	**BOP, kPa**	**Impulse, Pa·s**	**BOP, kPa**	**Impulse, Pa·s**	**BOP, kPa**	**Impulse, Pa·s**
**Experiment**	152±6	271±11	238±8	415±13	314±25	556±40
**ConWep**	160	287	240	407	325	575
**TNT mass, kg**	19.8		34.2		66.0	
**Standoff distance, m**	7.0		7.0		7.6	
